# Aneurysm of the Abdominal Aorta

**Published:** 1907-12

**Authors:** 


**Aneurysm of the Abdominal Aorta.** By F. P. NUNNELEY, M.D., M.A. Pp. vii., 121. London: Baillière, Tindall & Cox. 1906.

The author has published this monograph, originally written as a dissertation for the M.D., partly at any rate at Professor Osler's suggestion. It consists of a careful analysis of thirty-two cases, the clinical and pathological notes of which were found in the records of St. George's Hospital; and though little is unearthed that has not been taught before, yet the book is of much value in several ways. It is in the first place a check on the statistical articles already published by Browne, Bryant and others. Again, several interesting ætiological data are brought into prominence, notably the rarity of the condition in women and the comparatively early age at which its victims are affected (between 25 and 45 in 75 per cent.). Only thirteen of the cases were correctly diagnosed during life, and some of Nunneley's observations on diagnosis are therefore of peculiar interest; in particular there is his remark that radiography is only of value in confirming a positive diagnosis. Perhaps the most important section is that dealing with treatment. It is naturally pessimistic in tone, perhaps unduly so in the paragraph dealing with potassium iodide. Lancereaux's gelatin treatment is also described as unsatisfactory. The results of surgical treatment, bad as they have been hitherto, yet leave room for hope as to the future. Apart from its intrinsic worth, this monograph is an eloquent testimony to the value of honestly-kept records, whether clinical or pathological; and its author is to be congratulated from all points of view upon this solid addition to our knowledge of an interesting and important subject.

